# Multiple Estimates of Transmissibility for the 2009 Influenza Pandemic Based on Influenza-like-Illness Data from Small US Military Populations

**DOI:** 10.1371/journal.pcbi.1003064

**Published:** 2013-05-16

**Authors:** Pete Riley, Michal Ben-Nun, Richard Armenta, Jon A. Linker, Angela A. Eick, Jose L. Sanchez, Dylan George, David P. Bacon, Steven Riley

**Affiliations:** 1 Predictive Science Inc., San Diego, California, United States of America; 2 Armed Forces Health Surveillance Center, Silver Spring, Maryland, United States of America; 3 National Center for Medical Intelligence, Fort Detrick, Maryland, United States of America; 4 Science Applications International Corporation, McLean, Virginia, United States of America; 5 MRC Centre for Outbreak Analysis and Modelling, Imperial College London, London, United Kingdom; University of New South Wales, Australia

## Abstract

Rapidly characterizing the amplitude and variability in transmissibility of novel human influenza strains as they emerge is a key public health priority. However, comparison of early estimates of the basic reproduction number during the 2009 pandemic were challenging because of inconsistent data sources and methods. Here, we define and analyze influenza-like-illness (ILI) case data from 2009–2010 for the 50 largest spatially distinct US military installations (military population defined by zip code, MPZ). We used publicly available data from non-military sources to show that patterns of ILI incidence in many of these MPZs closely followed the pattern of their enclosing civilian population. After characterizing the broad patterns of incidence (e.g. single-peak, double-peak), we defined a parsimonious SIR-like model with two possible values for intrinsic transmissibility across three epochs. We fitted the parameters of this model to data from all 50 MPZs, finding them to be reasonably well clustered with a median (mean) value of 1.39 (1.57) and standard deviation of 0.41. An increasing temporal trend in transmissibility (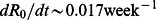, p-value: 0.013) during the period of our study was robust to the removal of high transmissibility outliers and to the removal of the smaller 20 MPZs. Our results demonstrate the utility of rapidly available – and consistent – data from multiple populations.

## Author Summary

The ability to rapidly and reliably characterize novel strains of influenza in terms of their transmissibility is crucial for health planners: without good estimates for key parameters it is not possible to identify the appropriate strength of interventions or the spatial optimization of interventions based on variability in transmissibility. While the transmission of influenza in civilian societies has been relatively well-studied, it has received considerably less attention within military populations; yet the consequences, particularly during wartime, are arguably far greater. We have investigated the incidence for the 50 largest military installations in the USA, and, to the extent possible, compared them with the profiles of the enclosing civilian populations during the 2009 influenza pandemic. We infer that the local civilian population drove the timing of peak incidence at the military installations. We also developed and applied a two-peak SIR model to capture the essential properties of the pandemic at each installation, finding that transmissibility tended to increase during the course of the pandemic.

## Introduction

Novel strains of influenza emerge as pandemics either from animal reservoirs [1] or from reassortment in humans [2]. Pandemic strains are characterized by low levels of population immunity that permit higher levels of incidence. However, pandemic strains are not necessarily intrinsically more transmissible nor more severe (at the individual level) than the previously circulating seasonal strains they often replace [3]. An ability to rapidly and reliably characterize novel strains in terms of their transmissibility is crucial for health planners in both the civilian and military domains: without good estimates for key parameters it is not possible to identify the appropriate strength of interventions [4], nor is it possible to consider the spatial optimization of interventions based on variability in transmissibility [5].

The basic reproduction number, 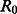, quantifies the transmissibility of a pathogen and is defined to be the average number of secondary cases generated by one infectious individual in an otherwise susceptible population [6]. Pathogen-population combinations with 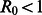 typically do not generate large outbreaks following an introduction (although they may generate self-limiting stuttering chains of transmission [7]). The efficacy of an intervention can be measured by the proportion of transmission it is able to avert, thus, high-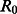 pathogen-population combinations such as measles in sub-Saharan Africa [8] require highly effective intervention campaigns in order to achieve control. However, although the main utility of 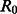 is often thought to be in quantifying the strength of intervention needed for control, it is also important in determining the likely efficacy of mitigating interventions [9] in reducing the number of infections [10] when control cannot be achieved.

Although often assumed to be a universal constant for a particular pathogen, 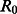 is variable across time and population for a variety of scales: the 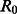 within an elementary school may be different from the 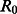 within a nearby high school in the same way that the 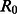 within one northern hemisphere city infected during September may be different from the 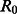 within a second city infected during January. Early estimates of 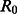 in civilian populations during the 2009 pandemic ranged from 1.1 to 3.3 and were based on influenza-like-illness data from an ad hoc data gathering process in a single population [11]–[15]. This wide range of values could be explained by one or more of: intrinsic differences between populations, such as host immunity or predisposition to infection; modifications in behavior over time, such as increased or decreased hand hygiene; seasonal climatic variability; methodological differences in parameter estimation; variability in pathogen-specific virulence across regions and/or time; and variability in underlying data-gathering processes.

Crucially, because the 2009 pandemic strain was mild, substantive policy uncertainty did not arise from this discrepancy: there was no need to choose between available mitigating interventions because costly strategies were not justified. Nonetheless, should the next emergent human influenza strain be more severe, any estimate of the absolute benefits of transmission blocking interventions would be highly sensitive to variation in 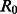 of the scale seen in the literature from the 2009 pandemic. Therefore, prior to the start of the next pandemic, there is clear public health value in the timely coupling of routinely collected high-quality data with robust parameter estimation. Such systems could be calibrated each year using data from seasonal influenza epidemics, and would provide useful decision support during severe non-pandemic influenza seasons.

In this study, we use data from the Defense Medical Surveillance System (DMSS) to: (1) describe the pandemic profiles observed at military installations; (2) compare them with available data from the surrounding civilian population to evaluate how much civilian populations drive incidence in military installations; and (3) use a parsimonious transmission model to estimate installation-specific 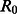 values. In addition to allowing us to characterize military-specific patterns, our study offers potential insights into their surrounding civilian populations. Possible strengths of analyses based on the DMSS data compared to other data sources for civilian population are: localization (to within a zip code); consistent reporting over many years; and, potentially, near-realtime availability.

## Methods

### Data

We obtained data from the Armed Forces Health Surveillance Center (AFHSC) consisting of outpatient visits to permanent military treatment facilities (MTFs) by active duty military personnel for a range of ICD (international classification of diseases)-9 codes associated with respiratory-related illnesses between January 1, 2009 and April 30, 2011. For each record, the data contained: a unique study identifier for the individual; ICD-9 codes associated with that visit; and the zip code (5 digits) of the clinic location. We used the zip code of the reporting clinic as a proxy with which to define military installation: we do not explicitly represent military installations or bases, rather, we assume that case reports from the same zip code are from the same military installation. Each record (an anonymized Study ID) was assigned as either “ILI-large” (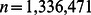) or “ILI-small” (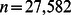) using a set of classifications based on ICD-9 codes [16]. The definition of ILI-large was broader and included non-specific diagnosis such as ‘viral infection’ and ‘acute nasopharyngitis’ (Table S2). The definition of ILI-small was more constrained and included: ‘Influenza w/other respiratory manifestations’ (25,293), ‘Influenza with manifestation not elsewhere classified (NEC)’ (1006), ‘Infectious upper respiratory, multiple sites, acute NEC’ (897), and ‘influenza with pneumonia’ (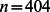). See Table S2 for further details. We further trimmed the data temporally to cover the period from April 1, 2009 through June 1, 2010, and ranked these installations by size according to the total number of ILI-small cases they reported. Although the AFHSC DMMS data includes clinic visits by military personnel at many locations around the world, here we focus on the top-50 largest profiles, 47 of which, were located within the USA. Of the remaining three, one was located in Landstuhl, Germany, and two were located in Japan (Misawa and Yokosuka).

We obtained civilian data through a variety of means. County-level data were generally acquired directly from the appropriate public health services department or from the CDC. CDC ILI data were obtained from the flu activity and surveillance website [17].

### Models

We considered a set of independent deterministic transmission models, one for each military installation. For each, we solved the following set of equations:
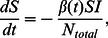
(1)

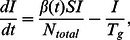
(2)

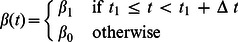
(3)where 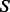 represents the number of susceptible individuals, 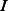 is the number of infectious individuals, 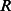 is the number of recovered individuals, and 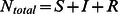 is the total active duty population size at each installation.

The incidence (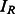) is given by 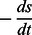, which computationally, is estimated by:
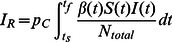
(4)where 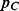 is the proportion of the infectious active duty population that present themselves to a clinic with ILI-small symptoms, and the integral runs over a week from 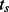 to 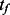.

The total population at each military installation, 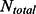, is arguably a militarily-sensitive parameter. For this study, we estimated it using publicly-available data in the following way. First, we calculated the total number of active duty out-patient visits at each installation for all causes over the period from January 2009 through April 2011, 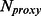, which we suggest is proportional to the total population at each installation. To estimate the coefficient of proportionality, 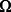, we identified a subset of the installations for which reasonably reliable estimates for the total population have been published (Figure S3). Estimates of 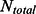 for the top-50 installations are shown in Table S3, Column 3.

The time-dependent term, 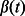, changes from 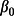 to 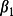 at time 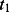 and returns to 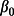 after an interval 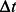. Equivalently, we allowed 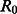 to change at some point in time, 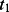, to a new value 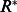. Intuitively, this definition makes sense if we imagine some mechanism, such as school closures on installations, the deployment of troops, or some other behavior modification to drive the effective contact rate down, and, hence, 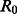. For purposes of generality, however, we did not impose any requirement that 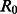 decrease at this time.

Even during a pandemic, there are reasons other than influenza infection for cases to present as ILI-small. Therefore, we also included a noise term. It was implemented as a constant added to the model output for incidence during the optimization procedure, resulting in a total of eight parameters. For fitting purposes we further trimmed the data in time from the outside inward and fit to all data bounded by the first non-zero values.

Following [18] we define the Akaike Information criterion (AIC), which is a measure of the relative goodness of fit of a model, for a single model at the 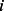
th military installation to be
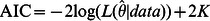
(5)where 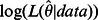 is the value of the maximized log-likelihood over the unknown parameters (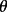), given the data and the model (Text S1). When the total number of parameters (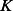) is large relative to the sample size (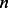), the reduced Akaike Information Criterion is preferred:
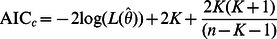
(6)


Model fits were optimized by first defining a multidimensional hypercube, running the model simulations with the hypergrid parameters and ranking the resulting 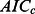 scores. Each of the top 1,000 scores is then used as an initial guess for a multi-dimensional Nelder-Mead (also known as downhill simplex) minimization of the Log-Likelihood. The lowest value of these searches is reported. The bounds and resolution of the hypercube are given in Table S1. We note in passing that while the results presented here relied on pseudo-Poisson log-likelihoods, we also used both 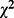 and least-squares fits methods to optimize the solutions with no significant differences in results.

## Results

**Figure 1:**
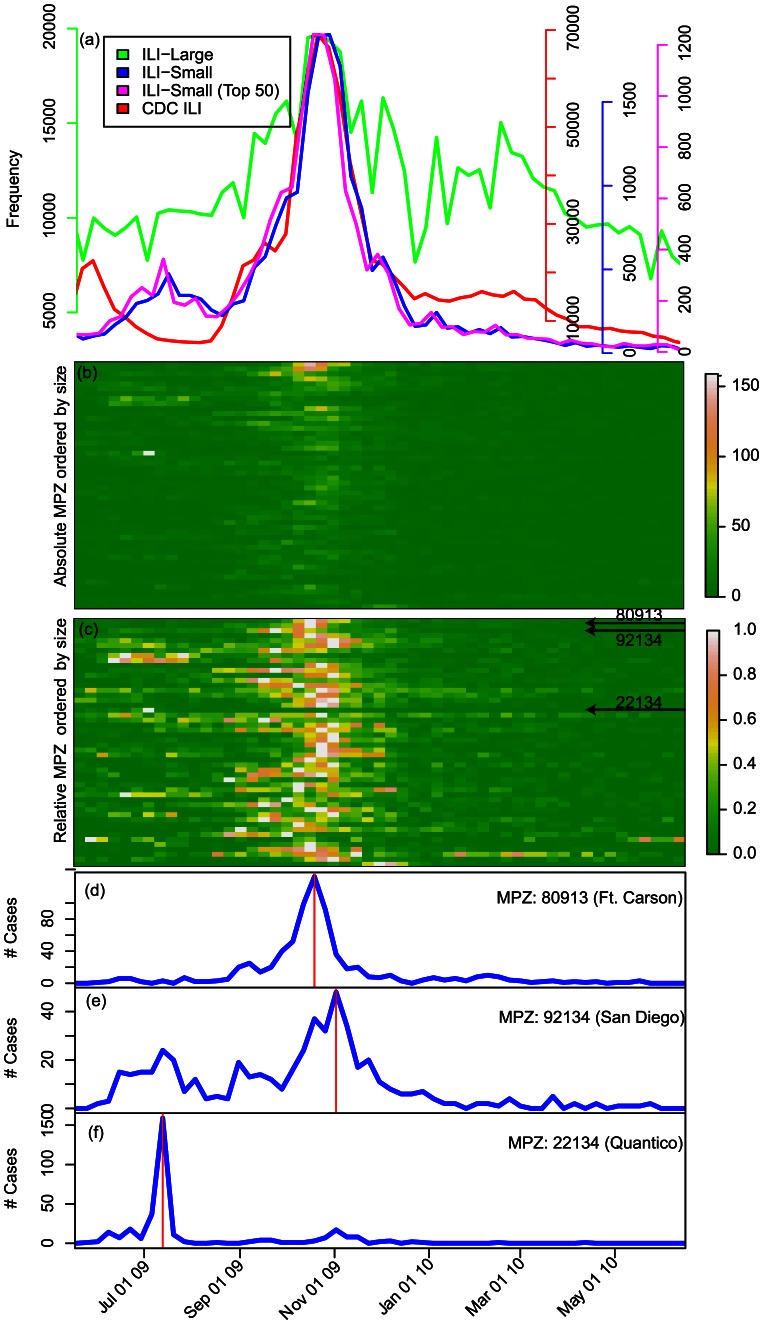
Reported cases at US military installations during the 2009 influenza pandemic. **a** number of reported cases per week of: ILI-large (green); ILI-small (blue); the top 50 military installations' contribution to ILI-small (magenta); and the CDC's ILI weekly surveillance (red). Profiles overlap because of the independent y-axis scaling. **b** heat map representation of ILI-small profiles for each of the top 50 military installations by zip code (MPZ), ordered by total number of ILI-small reported (largest at top). **c** as **b** but each profile has been renormalized to its own maximum value, thus, highlighting relative variations. Incidence curves for: Fort Carson **d**, just outside of Colorado Springs in El Paso County, Colorado (MPZ 80913), containing over 21,000 soldiers; Bob Wilson Naval Hospital **e** in San Diego, which serves as a clinic for several military installations primarily within San Diego County, and including MCAS Miramar (MPZ 92134); and Marine base at Quantico, Virginia(MPZ 22134) **f**, which is a major training facility for both Marines and federal law enforcement agencies. The timing of individual MPZ peaks is marked by the red vertical line. A complete set of the profiles for the largest 50 MPZs is given in Figure S1.

We compared time series for both ILI-small and ILI-large with available civilian data from the Centers for Disease Control and Prevention (CDC, www.cdc.gov, Figure 1a) for the time period between April 1, 2009 and March 31, 2010. There was substantially greater temporal correlation between the CDC time series and ILI-small (Pearson correlation 0.91) than with ILI-large (Pearson correlation 0.80). The time series for ILI-small cases arising from the largest 50 military installations (as defined in Materials and Methods) was similar to the total time-series in both trend and amplitude (Figure 1a). For the same time period (April 1, 2009 through March 31, 2010), 13,690 out of 21,285 ILI-small cases (64%) in the DMSS data occurred in the largest 50 installations. Therefore, we restrict ourselves to ILI-small for the remainder of this study.

The aggregate pattern of incidence of ILI-small for the largest 50 installations was driven both by qualitative variation in the shape of incidence curves and by variation in the timing of epidemic peaks (Figure 1b). Broadly speaking, the shape of each installation incidence curve could be described as: (1) a typical single-peaked epidemic profile, that is, consisting of a single exponential rise, peak, and more gradual decay; (2) a bimodal profile, consisting of two peaks separated by a month or two; (3) a very narrow, sharp peak, where the entire outbreak is complete within ∼4weeks; or (4) a prolonged, noisy, and relatively flat profile, often containing a single-peaked profile within it. For example, the military populations defined by zip code (MPZ) 80913 (Colorado, MPZ-80913) experienced a classic epidemic profile for the incidence of ILI-small; taking off in early September, peaking in the middle of November and then dropping to low levels by early January (Figure 1c). In contrast, the profile at MPZ-92134 (southern California, Figure 1d) displayed two clear peaks, one in July and another at the end of October 2009. Finally, at MPZ-22134 (Quantico, Virginia) a single, sharp peak was observed in July, with only the hint of a second wave in early November (Figure 1e). The variability of the profiles for the top 50 MPZs is summarized in the heat chart of Figure 1, which illustrates the variation in timing of the peaks. Individual line plots of incidence for each of the top 50 MPZs are shown in Figure S1.

**Figure 2:**
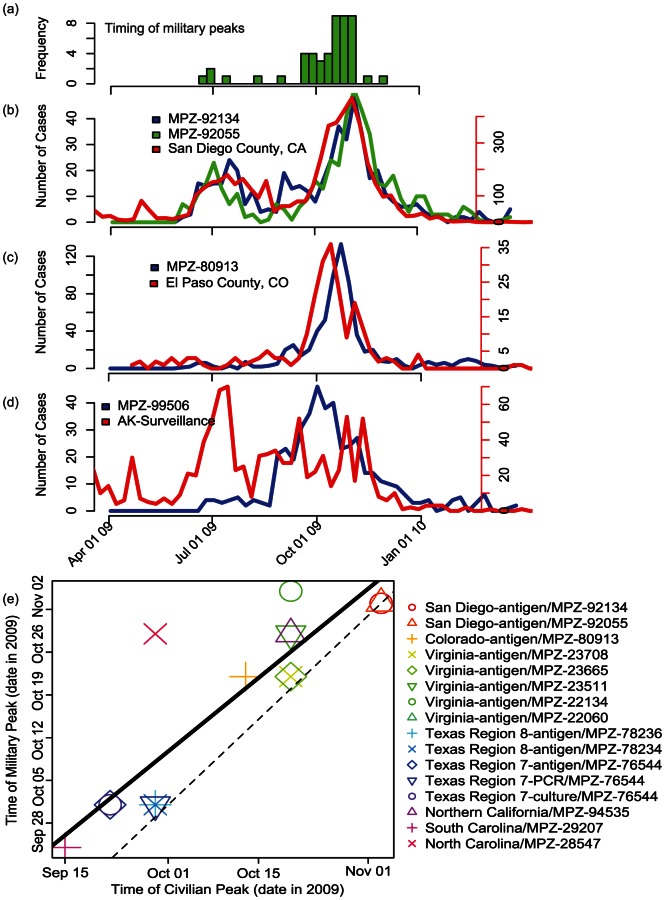
The timing of the pandemic peaks for military installations by zip code (MPZ) and their relationship to civilian profiles. **a** distribution of the timing of the peaks at each installation during the interval between April 1, 2009 and January 1, 2010. A number of installations showed evidence for two waves, one in the summer and one in October. Here, only the highest peak from the entire interval is shown. Comparison of military and civilian population profiles for three locations: **b** Incidence profiles for San Diego County, together with MCAS Miramar (MPZ 92134) and Camp Pendleton (MPZ 92055) bases; **c** El Paso County and Fort Carson Army Base (MPZ 80913); and **d** Alaska State (data at Borough/County level not available) and Elmendorf Air Force Base (MPZ 99506). **e** comparison of the timing of the peaks within MPZs and the nearest civilian populations for installations for which relatively localized civilian data could be obtained. The legend summarizes the type of civilian data obtained (confirmed/antigen, PCR, or culture) and the installation to which it was compared. The solid line is a linear regression to the data with a Pearson correlation coefficient of 0.9. Points lying above and to the left of the dashed line (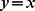) represent cases where the military peak lagged the civilian peak.

The peak weeks of incidence during 2009 for individual military installations were clustered primarily around one point during early Autumn 2009, with a few installations peaking as early as June 2009 (Figure 2a). The timing of peak weeks was not obviously correlated with longitude, latitude, average temperature, precipitation, or with distance from any of the known points of origin for the pandemic strain in the United States (Figure S2). However, for most military installations for which detailed civilian surveillance data were available for the region containing that zip code, there was a close correspondence between both the timing of the peak of the epidemic in the civilian population and the more detailed incidence profile in those civilian populations (Figure 2b and 2c). In a small number of cases, however, there was a relatively poor correlation (Figure 2d, see Discussion).

For each civilian/military profile pairing, we computed temporal cross correlations for the period from April 1st, 2009 through March 31, 2010. The correlation coefficients ranged from effectively zero (MPZ-22134) to 0.91 (MPZ-92134), although all but one were 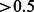. Moreover, the lag that maximized the correlation was typically one week. Thus, the profiles at the military installations were similar in structure to the civilian peaks but delayed by approximately one week (Figure 2e). We calculated an overall Pearson correlation coefficient of 0.89 between available pairs of civilian and military populations for the week of peak incidence. Some populations were used more than once in the calculation of the Pearson coefficient because multiple civilian datasets were available for individual military populations.

Our modeling framework permitted two alternate models (one nested within the other, see Methods) to estimate the transmissibility during the 2009 pandemic at each of the largest 50 MPZs: a one-peak model (four parameters) and a two-peak model (seven parameters). As would be expected, the 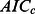 scores for the two-peak model were much better for MPZs that exhibited double peaks of incidence. However, we also found that the two-peak model always provided substantially better support for the data, even when the time series of incidence did not obviously show two separate peaks. Therefore, we report parameter values for the two-peak model for all military installations.

**Figure 3:**
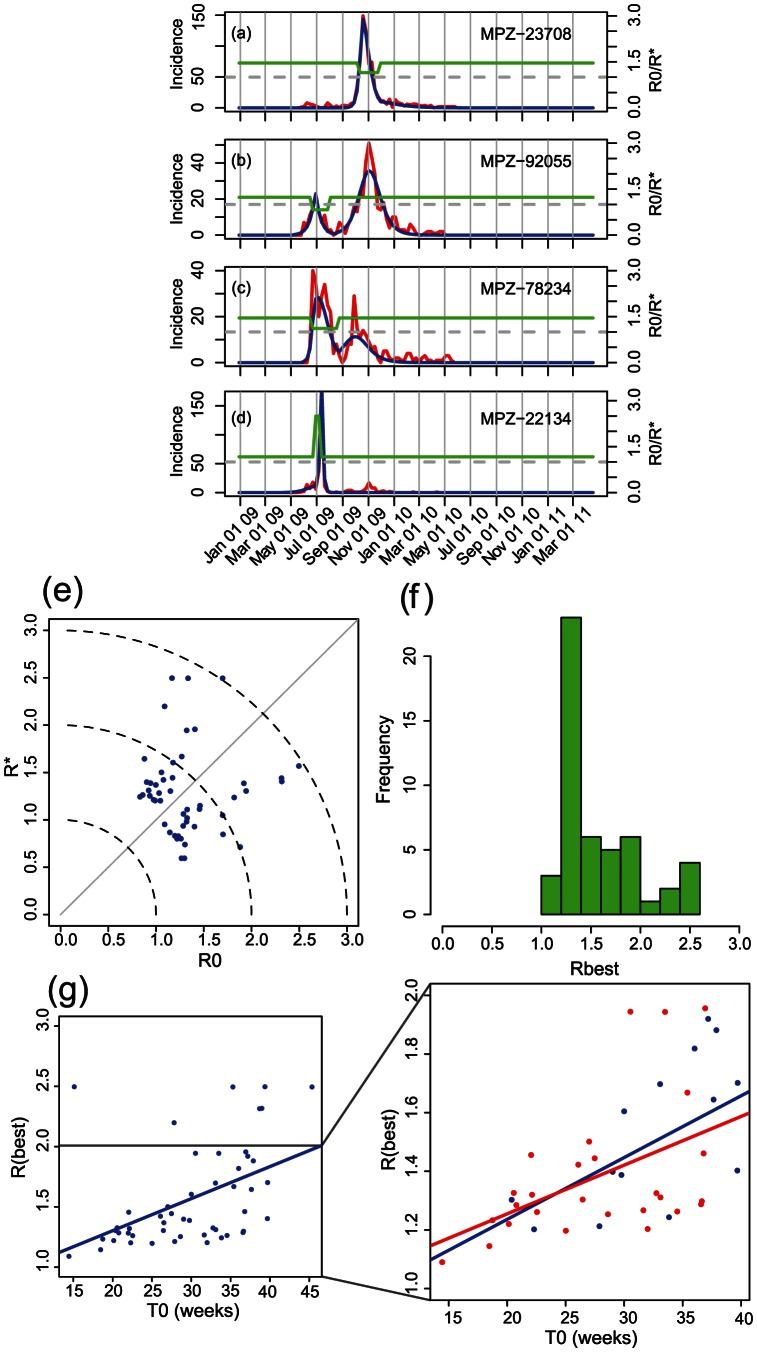
Model fits for the top 50 installations during the 2009 pandemic. (**a, b, c, and d**) Comparison of model fits with military installations for a selection of installations: (**a**) Portsmouth Naval Medical Hospital, Portsmouth, Virginia (MPZ-23708). This location produced the largest number of ILI-small cases. The hospital employs 4,300 active duty military and civilians but is also located near several Navy and Army facilities. The profile demonstrates a clean epidemic curve and the model fit closely matches the observed profile. (**b**) Camp Pendleton Marine base (MPZ-92055). The installation has five schools on the base, three of which fall under the Oceanside school district and two of which are managed by Fallbrook. (**c**) Fort Sam Houston Army base located in San Antonio, Texas (MPZ-78234). This large installation has over 70,000 family members, 15,000 retirees, and trains more than 25,000 students each year. An independent school district is located on the base. (**d**) Quantico Marine base (MPZ-22134). See Figure 2 for more details. In each panel a–d, the red line indicates data, the blue line indicates the model fit, and the green line shows the time evolution of 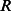. (**e**) Comparison of 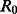 and 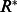 for the top 50 installations. The solid line marks a slope of one, while the dashed circular curves mark boundaries at 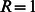, 2, and 3, serving to separate the outliers from the main cluster. (**f**) Distribution of 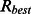, the maximum or 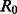 or 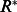, and the inferred value of 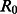 during the pandemic. The basic reproduction number clusters around a median value of 1.39 (mean 1.57); however, there are some notable exceptions. A complete set of model parameters is provided in Table S3 and histograms of 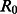, 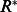, 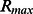 and 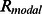 are shown in Figure S4. (**g**) The relationship between 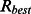 and the model-determined time of initial infection, 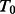. A linear regression to all fits (left) shows a modest increase in 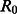 from the early summer to late fall. When the outliers (that is, 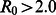) are removed from the analysis, the general rise in 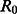 still persists. Moreover, when only the top 30 bases are included in the analysis (red points), the trend persists.

In general, we found satisfactory model fits to the military installations (Figure 3a–d, Table S1 and Figure S1). Usually, 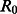 was estimated to be greater than 1, while 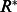 was less than 1. However, there were a number of exceptions (Figure 3e, Text S1, Table S3 and Figure S6). The fitted values of 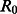 for the two peak-model model, when fitting to data from all 50 MPZs, were reasonably well clustered with a median (mean) value of 1.39 (1.57) and standard deviation of 0.41 (Table S4). We checked for any correlation between base size and our estimates of 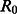 but did not find any (Figure S5).

In most of the two-peaked profiles, 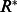 decreased at a point in time necessary to drive the initial wave downward, then returned to 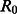 at the minimum between the two peaks, although this was not always the case. Moreover, for single-peaked profiles 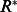 was used (by the model) prior to the main peak (e.g., Figure 3d), during the main wave (e.g., Figure 3a), or even following it (MPZ-22060, Table S3). Thus, it was not always obvious which single transmissibility parameter best captured the profile at each installation. To address this, we constructed an “optimum” estimate for 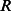, 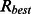, which was the maximum of 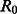 or 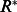 (Tables S1 and S3).

Seven installations had 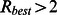: MPZ-23665 (Joint Base-Langley-Eustis); MPZ-22134 (Marine base at Quantico, VA (Figure 3d)); MPZ-85309 (Luke AFB); MPZ-29152 (Shaw AFB); MPZ-96319 (Misawa AFB); MPZ-57706 (Ellsworth AFB, SD); and MPZ-71459 (Fort Polk) (Table S3). It is interesting to note that a disproportionate number of these high-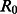 installations are Air Force bases, which, it could be argued, have the most civilian-like policies of any branch of the armed forces. In general, however, 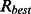 ranged from 1.0 to 2.0, being strongly biased toward the lower limit (Figure 3f).

During the course of the six months, over which the pandemic spread across the military installations, 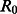 increased from 1.1 to 1.6 (Figure 3g, blue line). A least-squares fit to the data gave 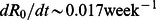, with a p-value of 0.013. The general increase was present both with and without the outliers and the trend was also captured by the top 30 installations as well as all 50.

## Discussion

In this study, we have derived incidence curves for individual MPZs using data from the AFHSC DMSS for the 2009 H1N1 pandemic. Comparison of the military incidence profiles with available civilian surveillance and testing data during the same time period suggested that most MPZs were temporally well-synchronized with their enclosing civilian population, but, importantly, tended to lag it by approximately one week. If we assume that the military installation is usually much smaller than the local civilian population, these findings suggest that the local civilian population is driving the timing of peak incidence in many military installations. Using SIR-like transmission models [19], we described a gradual increase in the transmissibility of influenza during 2009 in these populations.

Our study employed a number of assumptions that require careful consideration. First, we estimated the total population at each installation (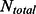) by assuming that the total number of visits to a clinic for all causes was a reasonable proxy for the total number of active duty personnel at that location (Figure S3). We estimated the constant of proportionality by comparing this number to published base sizes. However, in addition to intrinsic inaccuracies that these numbers may have, they are also subject to change over time as troops are recruited, deployed, and/or base sizes are changed. Fortunately, these “denominator” data, while undoubtedly sensitive information, are likely well known by military planners. Thus, in the hands of military personnel, these analysis could be easily re-run with significant improvement.

Second, our analysis also assumed a constant value for 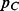, the proportion of infectious individuals that presented themselves to a clinic. This assumption was made for simplicity, enabling us to address the fundamental properties of the incidence profiles and estimate 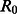. 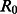 is clearly a key parameter that needs to be estimated early in an outbreak to guide policy makers in what types of intervention strategies, if any, should be employed. However, 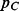, which is a measure of the severity of the pandemic, is rapidly gaining appreciation. This will be addressed in a forthcoming study.

Third, we did not explicitly include age-dependent effects, rates of reporting, nor accurate estimates of the population at-risk, all of which could potentially improve the utility of this approach. However, given the more tightly clustered age distribution within the active component of the military (typically 18–45 years old), together with the smaller number of cases that would define each profile, we suggest that our fitted models have good utility for the characterization of transmissibility. However, with accurate age-specific denominator data for each population we are confident that these methods can be expanded to allow a more finely-resolved study of age-specific transmissibility.

Although on average there was good correlation between the military installations and the enclosing civilian populations, this was not universally true. For MPZs that did not track well with the surrounding population, credible explanations can be given. For example, at Elmendorf AFB, just outside Anchorage Alaska, while the profile on-base was relatively simple, the civilian curve was considerably more complex. Alaska's civilian population, however, is modulated substantially with tourists, which over the course of a year outnumber residents by a factor of two. The two installations in Japan – Misawa and Yokusuka – displayed peaks that coincided with the trailing portion of the bimodal Japanese pandemic, one at the end of each wave. It is possible that, here, military personnel were insulated from the civilian population earlier in each wave. In contrast, the installation at Landstuhl, Germany, provided the only example where a military installation peaked significantly earlier than the civilian population. Here, it is quite possible that the pandemic at the installation was brought by troops recently deployed there from the United States. Thus, the lack of synchronicity at foreign installations can be explained by the fact that such troops mix far less frequently with the surrounding populations. More generally, the analysis presented here could act as a starting-point for the development of more detailed models of different types of military populations and for the systematic identification of a subset of installations that act as accurate sentinels for nearby civilian populations.

Our estimate for the basic reproduction number (mean: 1.57, median: 1.38) is generally consistent with those found using various civilian data (e.g., [11], [20]–[27]), and is relatively clustered (quartiles: 1.27 and 1.79, see Table S4). One might have expected higher values, particularly at installations supporting new recruits, or with on-base families and schools, but this does not appear to be the case. Further, our analyses do not suffer from obvious population selection-bias, as is the case with many early-outbreak studies. Rather, these data originate from routine episode recording for health insurance purposes. Similar data-streams exist in the civilian domain but have less uniform spatial coverage and would be more difficult to make available in real time [28].

The trend for 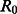 to increase with time is interesting in light of recent work on the seasonality of influenza transmission [29]. Although it is likely that media reports may have driven some individuals to seek treatment when they would otherwise not have done so, and that this effect varied over the course of our study, it would not have affected our description of trends in transmissibility to a large degree. Values for 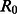 in our analyses were driven by the growth rate of incidence, not by the absolute level of incidence. Therefore, a gradual change in the propensity to report over many months would not affect our reported trend in 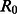. More rapid increases during the period of exponential growth at a specific base would affect our results and it is certainly possible that such changes in behavior may have occurred during late April 2009. However, we would expect those changes to bias 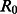 upwards during the early part of our study, which is not consistent with the pattern we report.

It is intriguing that the two-peak model consistently out-performed (based on AIC results) the one-peak model, even for profiles that visually appear to display a single, classic profile consisting of a sharp exponential rise, peak and slower decay. This suggests that even for these apparently straight-forward profiles, there may be some underlying mechanism at work that makes use of the freedom of the extra parameters. It is possible, for example, that changes in behavior or exchange of personnel may sufficiently modulate the basic profiles to the point that a seven-parameter model is appropriate. More generally, these results suggest subtle dynamics around the peak of short-time scale respiratory infections not captured by the very simple saturation process of the classic SIR model.

## Supporting Information

Figure S1 Influenza incidence (i.e, the number of reported ILI-small cases per week (red) and model fit (blue) as a function of time during the 2009 pandemic for the top-50 military installations. The value of the basic reproduction number is shown in green. A value of 1.0 is indicated by the dashed grey line. The military installations are ordered by the total number of ILI-small cases reported.
(PDF)

Figure S2Scatter plot matrix comparing the relative timing (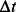) of the peaks at each of the top 20 installations with: the total number of ILI-small cases at each base (np); longitude (lon); latitude (lat); distance from MPZ-92134 (d92134, this is included for illustration - other potential ‘origins’ were also tested); average temperature (temp); and average precipitation (precip) at each installation. The red solid and dashed lines show linear regression and spread results, while the green line is a smoothed regression. The Figure was created using the “scatterplotMatrix” routine, which is part of the “car” R package.(EPS)

Figure S3Comparison of “N-PROXY” and “N-Web.” The quantity N-PROXY is the total number of outpatient visits for all causes to clinics serving a particular MPZ over a 2.3-year period from January 2009 through April 2011, which we anticipate is a proxy for the total number of personnel within each MPZ. “N-Web” is our estimate for the total number of troops at a selection of installations based on publicly-available sources (i.e., the web). These were limited to what we considered to be the most reliable values. This is obviously a subjective process, open to a number of sources of error and potential biases. To mitigate these, we employed a handful of heuristic rules, such as: (1) giving preference to descriptions that explicitly gave the number of active duty personnel; (2) omitting installations where the potential number was uncertain and/or fluctuated largely (navy installations serving ships, for example); and (3) giving additional weight to installations that were predominantly populated by a stable number of active duty personnel. Thus, to convert 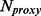 to 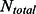 requires a constant of proportionality, 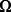, which we obtain by fitting the selected bases using: 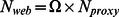. The best-fit line to the data, forcing the intercept to be zero, resulted in 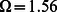.(EPS)

Figure S4Distribution of 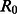, 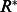, 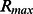, and 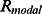. The first two parameters (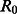, 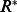) are estimated from the model fit. 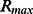 is the value of 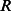 at the time of the peak in the model profile (not to be confused with 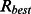, which is defined below). 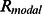 is the most common value of 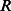 during the outbreak, weighted by the number of infectious individuals at each time. The two resulting distributions are quite similar, and even the two outliers (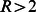) match. However, one issue remains: 15 (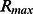) or 10 (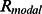) installations still produce estimates 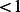. Since a pandemic must be associated with 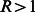, our final “best” estimate (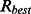) was defined to be the maximum of either 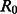 or 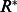. A complete set of model parameters is provided in Table S3.(EPS)

Figure S5Possible effects of bias from analyzing only the top-50 military installations. In terms of the total number of ILI cases, choosing the top 50 installations does not lead to bias in the sense that almost all of the cases are included in those installations and therefore, the estimates of 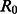 (and other parameters) are representative of the majority of the cases. We did not believe that the less-populated installations would produce as reliable estimates for the parameters and so did not include them in the analysis. If such a bias did exist, we might expect to see a correlation between military installation index (with 1 being the most populous installation and military installation 50 being the least) and, say, 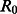. This panel suggests no obvious trend, and, thus, no evidence for any bias. On the other hand, we do note that the variability in 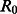 does increase modestly with smaller installations, which is what we would anticipate based on lower-number statistics and increased errors in the model fits.(EPS)

Figure S6Variation of model-determined basic reproduction number as a function of time during the 2009 pandemic. Each MPZ has been drawn in a different color and the corresponding zip code identified. In most cases, 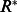 corresponds to a decrease during some portion (usually between the two peaks) of the profile. Less frequently, 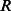 increases, often substantially, for a short period of time.(EPS)

Table S1 Minimum, maximum, and step size for hypercube parameters used in the study.
(PDF)

Table S2 ICD-9 Codes and frequencies for respiratory illnesses in the Defense Medical Surveillance System (DMSS) for the period January 2009 through April 2011.
(PDF)

Table S3 Model fit parameters for the top-50 MPZs.
(PDF)

Table S4Statistics for the values of 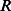 for the top-50 MPZs.(PDF)

Text S1 Methodology for estimation of goodness-of-fit.
(PDF)
