# Supplementary figures and images for: Multiple Estimates of Transmissibility for the 2009 Influenza Pandemic Based on Influenza-like-Illness Data from Small US Military Populations
Source: PLoS Comput Biol. 2013 May 16;9(5):e1003064. doi: 10.1371/journal.pcbi.1003064 (PMC3656103; doi:10.1371/journal.pcbi.1003064)

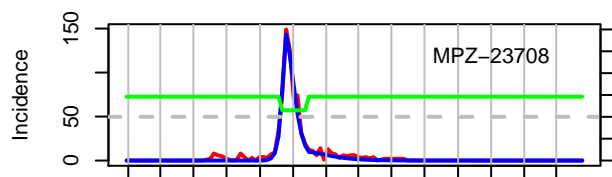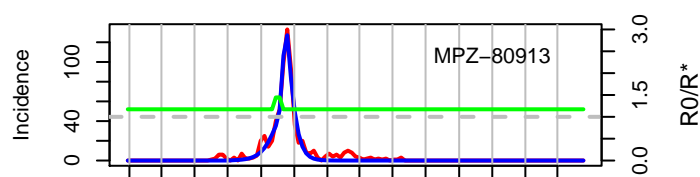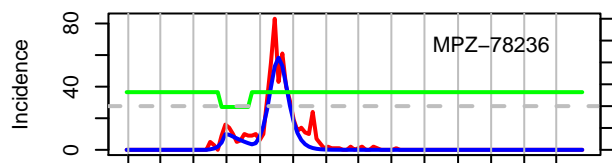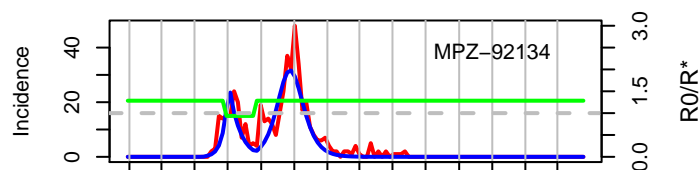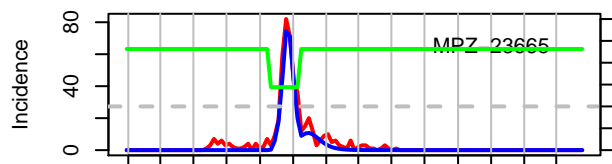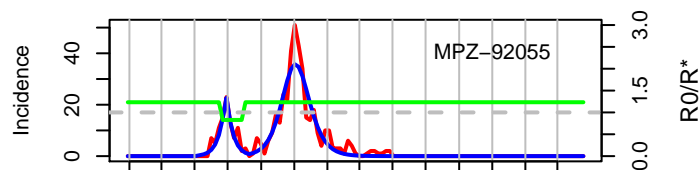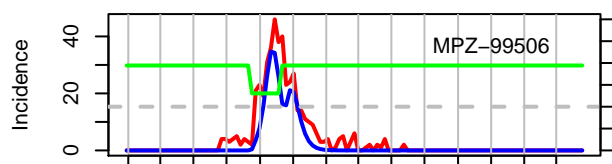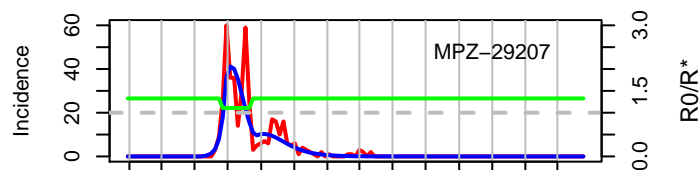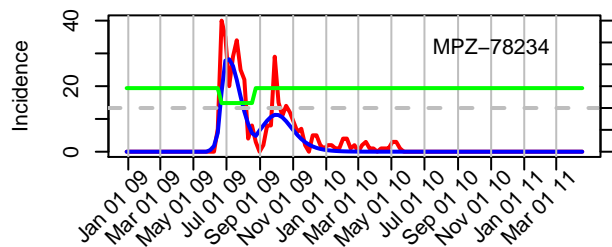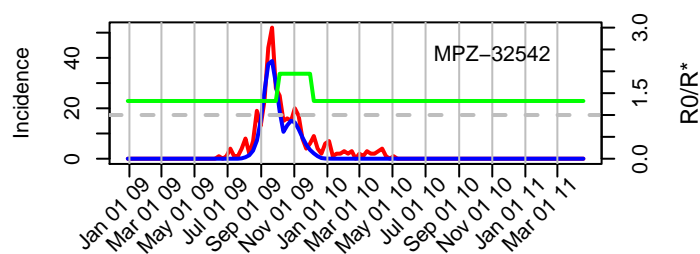

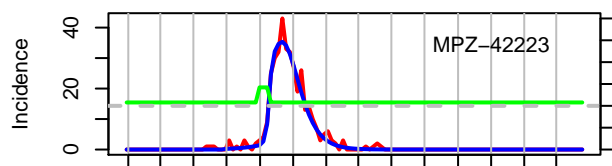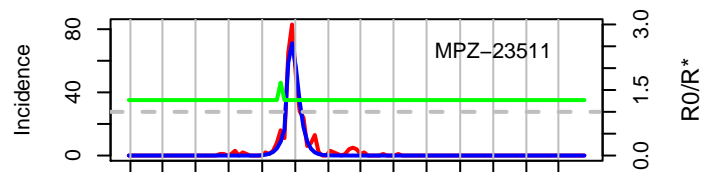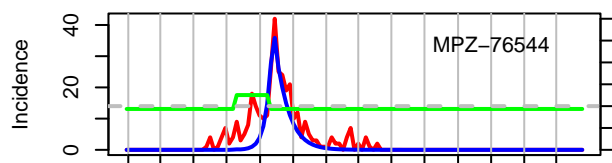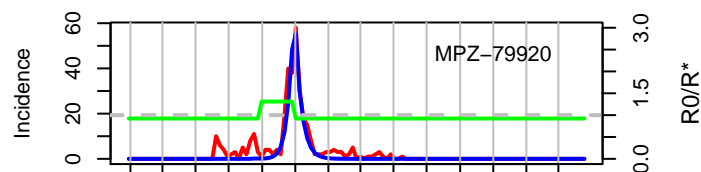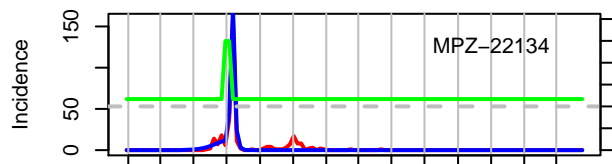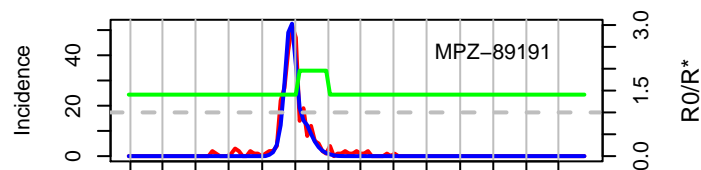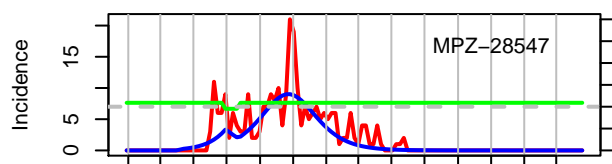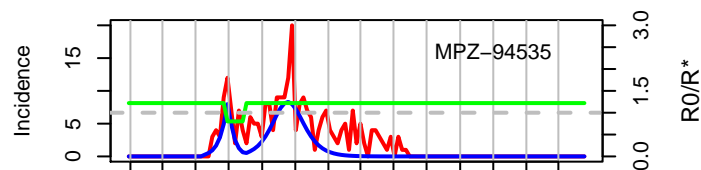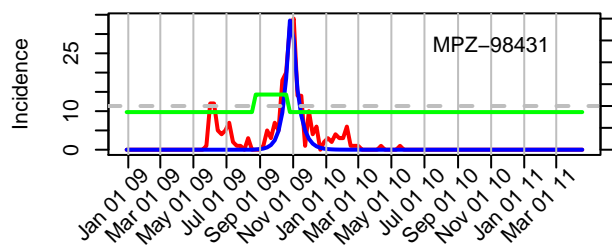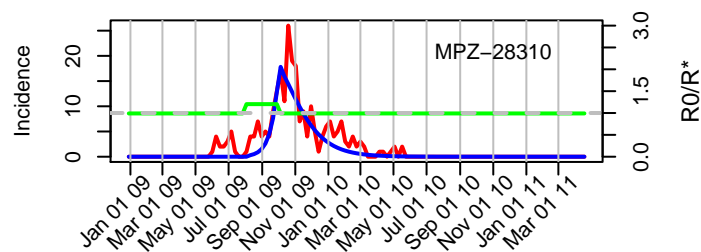

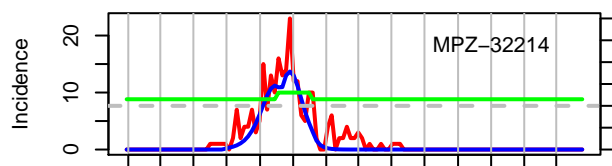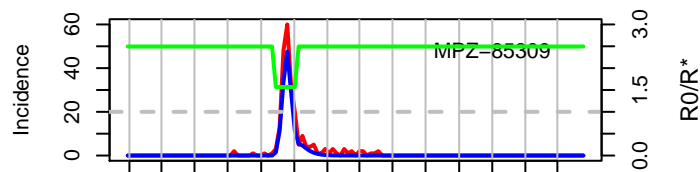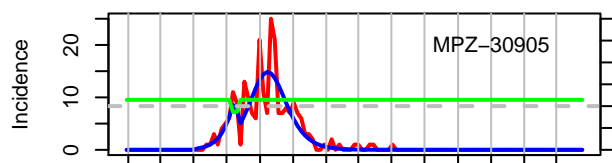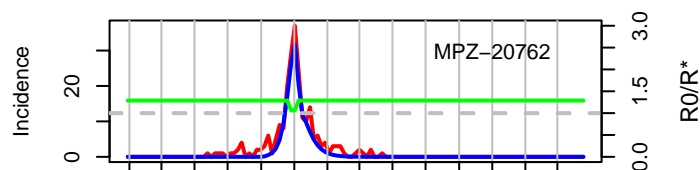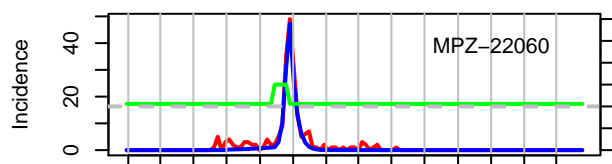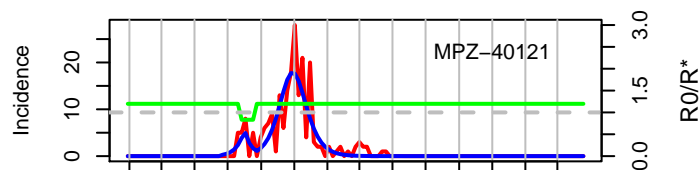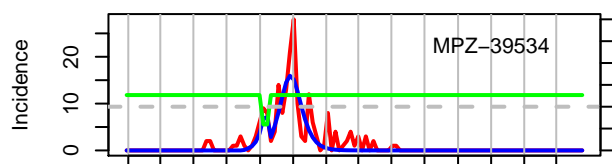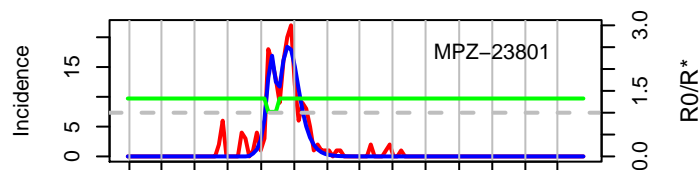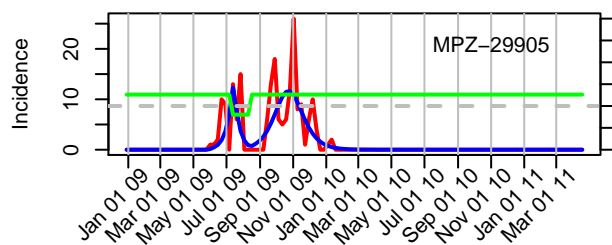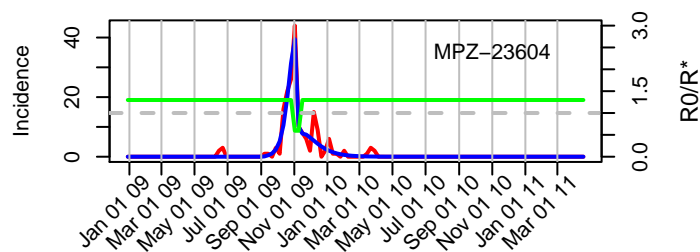

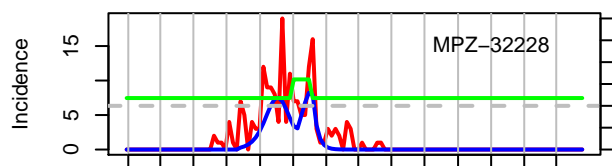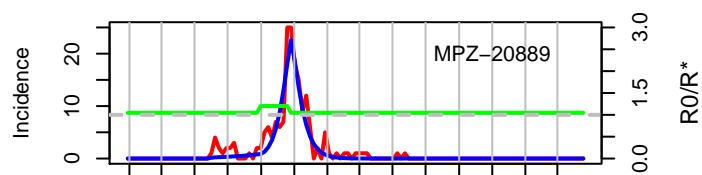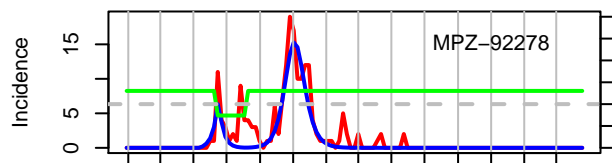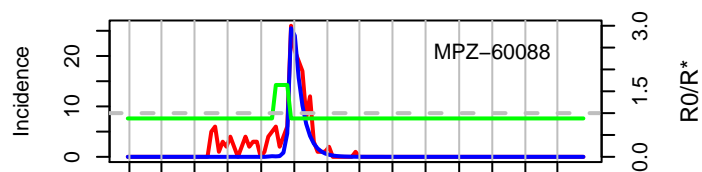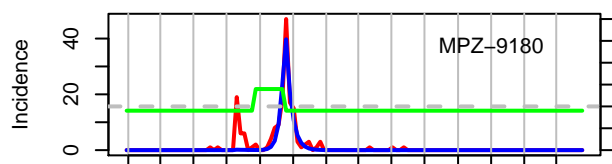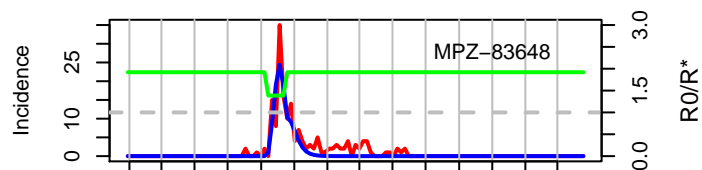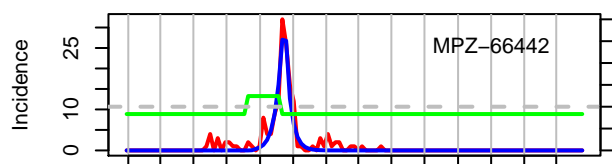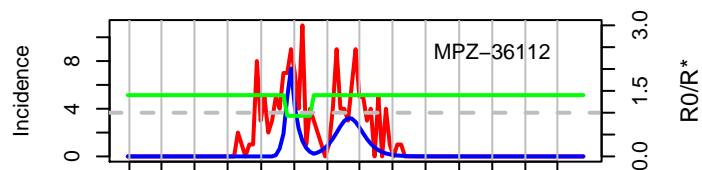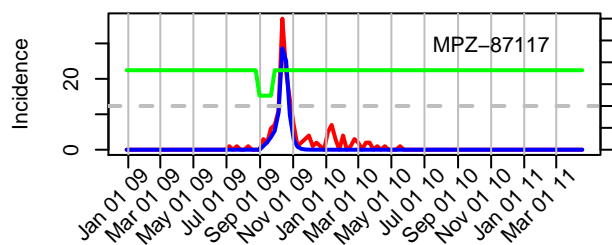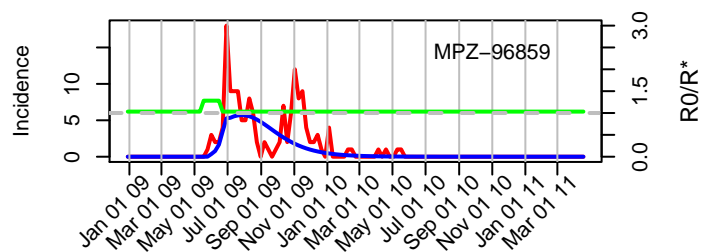

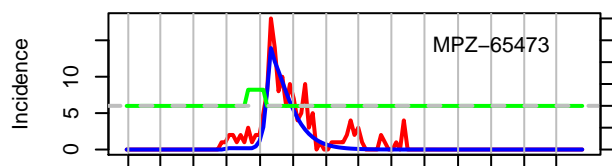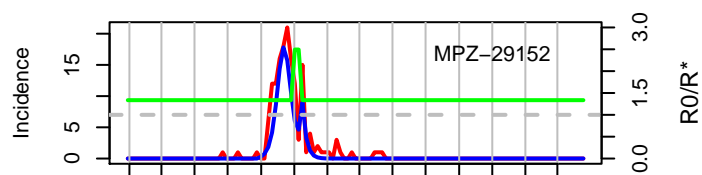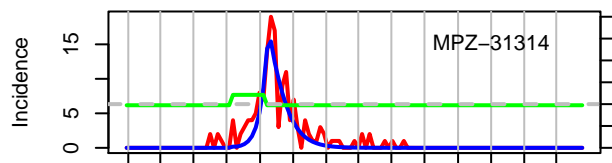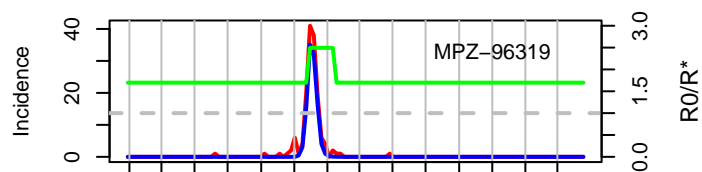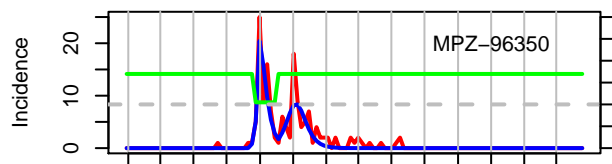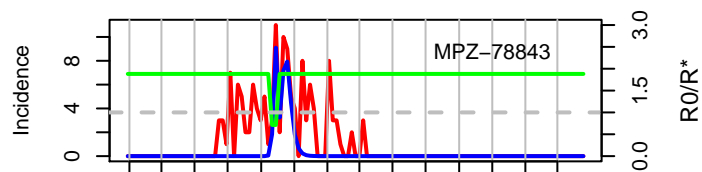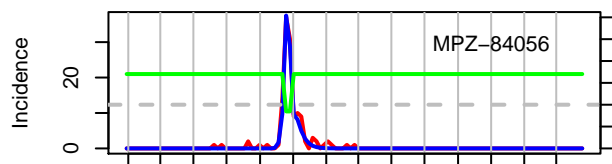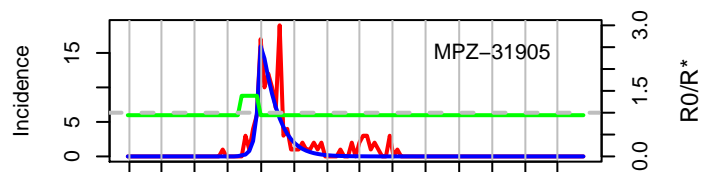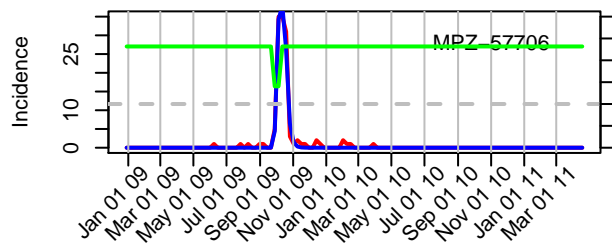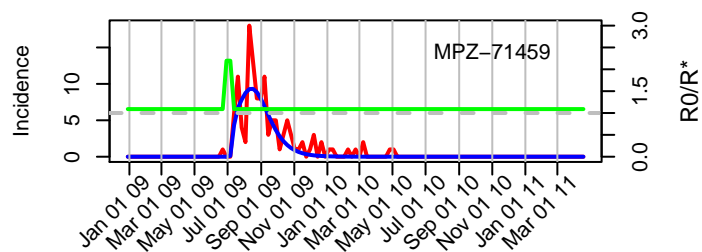

Supplement: Figure S1 — Influenza incidence (i.e, the number of reported ILI-small cases per week (red) and model fit (blue) as a function of time during the 2009 pandemic for the top-50 military installations. The value of the basic reproduction number is shown in green. A value of 1.0 is indicated by the dashed grey line. The military installations are ordered by the total number of ILI-small cases reported. (PDF) [file pcbi.1003064.s001.pdf]
