# Supplementary material for: Multiple Estimates of Transmissibility for the 2009 Influenza Pandemic Based on Influenza-like-Illness Data from Small US Military Populations
Source: PLoS Comput Biol. 2013 May 16;9(5):e1003064. doi: 10.1371/journal.pcbi.1003064 (PMC3656103; doi:10.1371/journal.pcbi.1003064)
Supplement: Table S1 — Minimum, maximum, and step size for hypercube parameters used in the study. (PDF) [file pcbi.1003064.s007.pdf]

Table S1 Hypercube parameters and step-size.

Parameter	min	max	step size
β_0 (weeks^{-1})	2	6	0.4
β_1 (weeks^{-1})	2	6	0.4
R_0	0.74	2.2	0.15*
R^*	0.74	2.2	0.15*
t_1 (weeks)	1	15	1
Δt (weeks)	0	8	1
baseline noise, ϕ_N	1	10	1

* Although the algorithm steps through β, since T_g is a constant, this is equivalent to stepping through R. Hence, we report both here.
