# Supplementary material for: Multiple Estimates of Transmissibility for the 2009 Influenza Pandemic Based on Influenza-like-Illness Data from Small US Military Populations
Source: PLoS Comput Biol. 2013 May 16;9(5):e1003064. doi: 10.1371/journal.pcbi.1003064 (PMC3656103; doi:10.1371/journal.pcbi.1003064)
Supplement: Table S2 — ICD-9 Codes and frequencies for respiratory illnesses in the Defense Medical Surveillance System (DMSS) for the period January 2009 through April 2011. (PDF) [file pcbi.1003064.s008.pdf]

**Table S2** ICD-9 Codes and frequencies for respiratory illnesses in the Defense Medical Surveillance System (DMSS) for the period January 2009 through April 2011.

| ICD-9 Code | Description | Number of Cases | ILI Group |
|---|---|---|---|
| 079.99 (7999) | Viral infection NOS | 158,105 | Large |
| 3829 | Unspecified otitis media, Otitis media: NOS, acute NOS, chronic NOS | 59,505 | Large |
| 460 | Acute nasopharyngitis | 165,375 | Large |
| 461.9 (4619) | Sinusitis, acute NOS | 60,709 | Large |
| 465.8 (4658) | Infectious upper respiratory, multiple sites, acute NEC | 897 | small |
| 465.9 (4659) | Upper respiratory infection, acute. NOS. | 491,810 | Large |
| 466 (4660) | Bronchitis, acute | 83,450 | Large |
| 486 (486) | Pneumonia, organism unspecified | 85,633 | Large |
| 487.0 (4870) | Influenza w/ pneumonia | 404 | Small |
| 487.1 (4871) | Influenza w/ other respiratory manifestations | 25,293 | Small |
| 487.8 (4878) | Influenza with manifestation NEC | 1006 | Small |
| 490 (490) | Bronchitis, not specified as acute or chronic | 85,526 | Large |
| 780.6 (7806) | Fever, nonperinatal | 56 | Large |
| 786.2 (7862) | Cough | 94,675 | Large |

1.0
