# Supplementary material for: Multiple Estimates of Transmissibility for the 2009 Influenza Pandemic Based on Influenza-like-Illness Data from Small US Military Populations
Source: PLoS Comput Biol. 2013 May 16;9(5):e1003064. doi: 10.1371/journal.pcbi.1003064 (PMC3656103; doi:10.1371/journal.pcbi.1003064)
Supplement: Table S3 — Model fit parameters for the top-50 MPZs. (PDF) [file pcbi.1003064.s009.pdf]

Table S3 Model fit parameters for the top-50 MPZs.

	MPZ^1	N^2_{total}	T^3_0 (weeks)	t^4_1 (weeks)	Δt^5 (weeks)	T^6_g (days)	Baseline[7]	p^8_C	R^9_0	$R_0(25)^{10}$	$R_0(75)^{11}$	R^{*12}	R^{13}_{modal}	R^{14}_{max}	R^{15}_{best}	AIC^{16}_c
1	23708	25376	36.79	5.61	6.39	2.60	2.15	0.07	1.46	1.13	1.20	1.15	1.15	1.15	1.46	284.32
2	80913	23400	27.48	13.11	1.77	2.60	3.00	0.07	1.17	1.18	1.29	1.44	1.17	1.17	1.44	308.46
3	78236	15359	22.13	4.77	7.47	2.60	2.40	0.07	1.32	1.20	1.24	0.98	1.32	1.32	1.32	339.39
4	92134	16445	20.78	6.64	7.05	2.60	2.09	0.07	1.28	1.15	1.21	0.94	1.28	1.28	1.28	301.05
5	23665	7697	38.69	0.89	7.30	2.60	2.70	0.07	2.31	1.42	1.52	1.44	1.44	1.44	2.31	261.06
6	92055	20181	18.74	8.09	5.95	2.60	1.15	0.07	1.23	1.15	1.19	0.83	1.23	1.23	1.23	257.30
7	99506	6139	33.50	1.09	7.70	2.60	3.15	0.07	1.94	1.30	1.35	1.30	1.94	1.30	1.94	299.96
8	29207	16770	20.56	6.32	7.36	2.60	0.83	0.07	1.33	1.08	1.18	1.11	1.11	1.11	1.33	287.51
9	78234	10492	22.05	4.08	8.35	2.60	1.20	0.07	1.46	1.09	1.21	1.11	1.11	1.11	1.46	288.46
10	32542	6521	30.54	11.42	8.52	2.60	2.40	0.07	1.32	1.29	1.35	1.94	1.32	1.32	1.94	235.41
11	42223	26974	26.08	10.28	2.66	2.60	0.80	0.07	1.08	1.06	1.14	1.42	1.08	1.08	1.42	181.58
12	23511	9548	35.41	6.54	0.91	2.60	1.02	0.07	1.27	1.34	1.36	1.67	1.27	1.27	1.67	214.58
13	76544	46822	28.62	2.24	8.53	2.60	3.20	0.07	0.94	0.89	1.02	1.25	0.94	0.94	1.25	283.79
14	79920	18111	33.11	3.01	8.28	2.60	2.35	0.07	0.92	0.98	1.19	1.31	0.92	0.92	1.31	269.47
15	22134	5621	15.12	12.28	1.63	2.60	1.80	0.07	1.17	0.75	1.65	2.50	1.17	1.17	2.50	306.43
16	89191	6152	36.93	9.29	7.98	2.60	0.86	0.07	1.41	1.35	1.38	1.96	1.41	1.41	1.96	179.02
17	28547	17935	14.45	12.57	3.19	2.60	1.62	0.07	1.09	0.85	1.04	0.95	1.09	1.09	1.09	263.42
18	94535	6008	20.14	6.90	4.34	2.60	2.67	0.07	1.22	1.08	1.16	0.80	1.22	1.22	1.22	245.78
19	98431	21546	34.54	1.09	8.52	2.60	2.41	0.07	0.86	0.89	0.99	1.26	0.86	0.86	1.26	297.71
20	28310	37270	32.01	0.00	8.50	2.60	1.60	0.07	0.99	0.96	1.05	1.20	0.99	0.99	1.20	238.32
21	32214	6591	26.45	15.07	8.10	2.60	1.60	0.07	1.15	1.16	1.19	1.30	1.15	1.30	1.30	229.35
22	85309	3229	39.37	0.77	5.39	2.60	1.09	0.07	2.50	1.64	1.66	1.57	1.57	1.57	2.50	152.23
23	30905	13780	18.48	10.77	1.50	2.60	0.80	0.07	1.14	1.11	1.13	0.87	1.14	1.14	1.14	209.69
24	20762	6327	36.58	8.42	1.66	2.60	1.20	0.07	1.29	1.25	1.27	1.06	1.29	1.06	1.29	182.90
25	22060	6474	27.01	13.06	3.43	2.60	1.06	0.07	1.06	1.07	1.34	1.50	1.06	1.06	1.50	184.46
26	40121	9244	25.01	6.58	3.20	2.60	0.80	0.07	1.20	1.15	1.18	0.83	1.20	1.20	1.20	215.53
27	39534	5428	31.65	5.85	1.06	2.60	1.20	0.07	1.27	1.19	1.21	0.59	1.27	1.27	1.27	233.72
28	23801	6067	32.76	5.56	2.13	2.60	0.80	0.07	1.33	1.20	1.21	1.02	1.33	1.33	1.33	188.60
29	29905	6916	22.55	6.50	4.33	2.60	0.90	0.07	1.26	0.68	1.18	0.80	1.26	0.80	1.26	240.76
30	23604	7438	36.64	8.39	1.07	2.60	0.80	0.07	1.30	1.14	1.25	0.59	1.30	0.59	1.30	177.84
31	32228	3273	30.00	15.53	4.27	2.60	1.61	0.07	1.18	1.17	1.19	1.60	1.18	1.60	1.60	220.11
32	20889	9974	22.30	13.81	7.88	2.60	0.80	0.07	1.05	1.05	1.16	1.20	1.05	1.05	1.20	190.74
33	92278	4660	20.38	4.67	7.10	2.60	1.19	0.07	1.30	1.15	1.23	0.74	1.30	1.30	1.30	229.86
34	60088	24302	37.65	2.83	3.55	2.60	1.95	0.07	0.88	0.85	0.89	1.64	0.88	0.88	1.64	188.36
35	9180	9031	29.02	6.53	7.11	2.60	0.97	0.07	0.90	0.85	1.19	1.40	0.90	0.90	1.40	257.98
36	83648	2534	37.18	1.59	4.22	2.60	1.60	0.07	1.92	1.40	1.44	1.39	1.92	1.39	1.92	187.45
37	66442	15634	33.84	0.00	8.53	2.60	0.98	0.07	0.83	0.82	0.90	1.24	0.83	0.83	1.24	179.78
38	36112	1998	39.68	3.78	6.38	2.60	2.37	0.07	1.40	1.13	1.20	0.93	1.40	0.93	1.40	214.01

39	87117	1831	36.02	0.84	3.67	2.60	1.20	0.07	1.82	1.56	1.59	1.24	1.82	1.82	1.82	186.67
40	96859	15475	21.99	0.00	4.35	2.60	0.80	0.07	1.03	0.95	1.06	1.28	1.03	1.03	1.28	259.26
41	65473	15146	26.51	7.46	4.46	2.60	1.05	0.07	1.00	0.96	1.00	1.37	1.00	1.00	1.37	183.77
42	29152	2719	35.29	10.34	1.05	2.60	0.80	0.07	1.34	1.32	1.35	2.49	1.34	1.34	2.49	153.51
43	31314	17657	27.89	1.89	8.41	2.60	0.81	0.07	0.97	0.92	1.01	1.21	0.97	0.97	1.21	186.18
44	96319	1899	45.35	4.16	6.29	2.60	0.80	0.07	1.70	1.71	1.72	2.49	2.49	2.49	2.49	139.79
45	96350	3103	33.07	2.67	5.01	2.60	0.81	0.07	1.70	1.02	1.36	1.05	1.70	1.05	1.70	174.92
46	78843	780	37.89	1.90	1.07	2.60	2.55	0.07	1.88	1.31	1.35	0.71	1.88	0.71	1.88	206.17
47	84056	2726	39.71	3.62	1.07	2.60	0.80	0.07	1.70	1.53	1.54	0.85	1.70	0.85	1.70	116.66
48	31905	25822	29.77	1.49	4.61	2.60	0.80	0.07	0.94	0.73	1.04	1.39	0.94	0.94	1.39	174.90
49	57706	1977	38.97	1.50	1.02	2.60	0.80	0.07	2.32	1.47	1.80	1.40	2.32	2.32	2.32	115.67
50	71459	7204	27.82	0.00	1.06	2.60	0.80	0.07	1.09	1.07	1.13	2.20	1.09	1.09	2.20	164.81

[1] MPZ = Military Installation by Zip Code. Anonymized individuals are identified by the zip-5 code of the clinic that they visited. Other possible locators would be: (1) the unit identifier code (UIC) of the unit the individual belongs to; (2) the zip-3 code of the UIC; or (3) the name (and/or location) of the installation to which their unit is assigned. However, these would likely introduce errors into our analysis, particularly when troops are temporarily transferred from one installation to another or troops are staged and/or deployed. MPZ, on the other hand, provides a measure of the instantaneous location of the individual when they present to the clinic with symptoms.

[2] N_{total} is the total population size for each MPZ, that is, the estimated number of individuals who are served by the clinics within a particular MPZ.

[3] T_0 is the initial time of onset of the epidemic in the model, that is, when the first susceptible individual becomes infectious. To within a week or two, it is also a measure of the timing of the model-fit peak.

[4] t_1 is the time at which R_0 changes to R^*.

[5] Δt is the duration over which the basic reproduction number remains at R^*.

[6] T_g is the generation time, or, in this model, the average time of infection. For the results shown here, T_g was fixed at 2.6 days.

[7] 'Baseline' is the amount of noise added to the solution during the fitting procedure and is optimized in the same manner as the other parameters. At the first time step, the number of infectious individuals is: $I = S \times p_{Inf} + seed_{Inf}$, where p_{Inf} is the probability of becoming infected and $seed_{Inf}$ is the baseline noise.

[8] p_C is the proportion of active military individuals who are infectious that visit a clinic.

[9] R_0 is the initial value of the basic reproduction number.

[10] $R_0(25)$ is the 25% quantile.

[11] $R_0(75)$ is the 75% quantile.

[12] R^* is the value that the basic reproduction number changes to at time t_1.

[13] R_{modal} is the value of R, either R_0 or R^*, corresponding to the most number of infections, that is, it is the value with the largest fraction of the area under the model fit profile.

[14] R_{max} is the value of R, either R_0 or R^*, at the time of peak model incidence.

[15] R_{best} is the maximum value of R_0 or R^*.

[16] AIC_c = Reduced Akaike Information Criterion. This is typically used when the number of parameters (K) is large relative to the sample size (n). [17] suggest using AIC_c unless $n/K > 40$. In our case, K is seven and n may be as large as one year, or 52 weeks, suggesting that $n/K < 7.4$, and AIC_c, not AIC should be used throughout.
