# Supplementary material for: Multiple Estimates of Transmissibility for the 2009 Influenza Pandemic Based on Influenza-like-Illness Data from Small US Military Populations
Source: PLoS Comput Biol. 2013 May 16;9(5):e1003064. doi: 10.1371/journal.pcbi.1003064 (PMC3656103; doi:10.1371/journal.pcbi.1003064)
Supplement: Table S4 — Statistics for the values of for the top-50 MPZs. (PDF) [file pcbi.1003064.s010.pdf]

Table S4 Statistics for the values of R for the top-50 MPZs.

	R_0	R^*	R_{modal}	R_{max}	R_{best}
mean	1.32	1.29	1.29	1.18	1.57
median	1.26	1.25	1.21	1.13	1.39
sd	0.39	0.46	0.36	0.34	0.41
10% quantile	0.93	0.80	0.93	0.86	1.21
25% quantile	1.06	0.96	1.06	0.98	1.27
50% quantile	1.26	1.25	1.21	1.13	1.39
75% quantile	1.41	1.44	1.33	1.30	1.79
90% quantile	1.89	1.95	1.82	1.45	2.31
