# Supplementary material for: Multiple Estimates of Transmissibility for the 2009 Influenza Pandemic Based on Influenza-like-Illness Data from Small US Military Populations
Source: PLoS Comput Biol. 2013 May 16;9(5):e1003064. doi: 10.1371/journal.pcbi.1003064 (PMC3656103; doi:10.1371/journal.pcbi.1003064)
Supplement: Text S1 — Methodology for estimation of goodness-of-fit. (PDF) [file pcbi.1003064.s011.pdf]

Text S1

Estimation of goodness-of-fit

We assume that the likelihood of a profile of observed incidence at some installation, $\{k_1, k_2, ..., k_i, ..., k_n\}$, where n is the total number of weeks over which the pandemic is observed, given a model with a set of parameters is equal to $\prod P(k_i|\lambda_i)$, running from 1 through n. Here, $P(k, \lambda)$ is the Poisson density:

$$P(k, \lambda) = \frac{\lambda^k e^{-\lambda}}{k!}, \tag{7}$$

where k is an integer equal to, or greater than zero and λ is a real positive number. The log of this is: $k \ln(\lambda) - \lambda - \ln(k!)$. Thus, the log-likelihood function we attempt to maximize for a given set of model parameters is:

$$PLL(\mathbf{k}|\mathbf{M}) = \sum_{i=1}^{n} k_i \ln(\lambda_i) - \lambda_i - \ln(k_i!) \tag{8}$$

where $\mathbf{M}$ are the parameters in the model that are fitted, and the sum runs over the time window over which we are fitting. For the four-parameter model, these are: (1) the average period of infection, T_g; (2) the basic reproduction number, R_0 (or, equivalently, the transmission rate, β_0); (3) the time of the initial infection, t_i; and (4) the effective number of susceptibles, N_{eff}. For the eight-parameter model, we add: (5) the value that R_0 is reduced, or increased to, R^* (or, equivalently, $\beta_0 \rightarrow \beta_1$); (6) the time at which this reduction occurs, t_1; (7) the duration over which the basic reproduction number is changed, Δt; and (8) a baseline noise component, ϕ_N. In the results presented here, we held $T_g = 2.6$ days, reducing the fit to seven parameters.
